# Some of the Latest Active Strengthening Techniques for Masonry Buildings: A Critical Analysis

**DOI:** 10.3390/ma12071151

**Published:** 2019-04-09

**Authors:** Elena Ferretti, Giovanni Pascale

**Affiliations:** Department of Civil, Environmental and Materials Engineering—DICAM, Alma Mater Studiorum Università di Bologna, Viale del Risorgimento 2, I40136 Bologna, Italy; giovanni.pascale@unibo.it

**Keywords:** retrofitting, earthquakes, masonry, historical buildings, active reinforcement, Mohr’s circles, CAM system, Φ system

## Abstract

The present paper deals with the retrofitting of unreinforced masonry (URM) buildings, subjected to in-plane shear and out of-plane loading when struck by an earthquake. After an introductive comparison between some of the latest punctual and continuous active retrofitting methods, the authors focused on the two most effective active continuous techniques, the CAM (Active Confinement of Masonry) system and the Φ system, which also improve the box-type behavior of buildings. These two retrofitting systems allow increasing both the static and dynamic load-bearing capacity of masonry buildings. Nevertheless, information on how they actually modify the stress field in static conditions is lacking and sometimes questionable in the literature. Therefore, the authors performed a static analysis in the plane of Mohr/Coulomb, with the dual intent to clarify which of the two is preferable under static conditions and whether the models currently used to design the retrofitting systems are fully adequate.

## 1. Introduction

**Figure 1:**
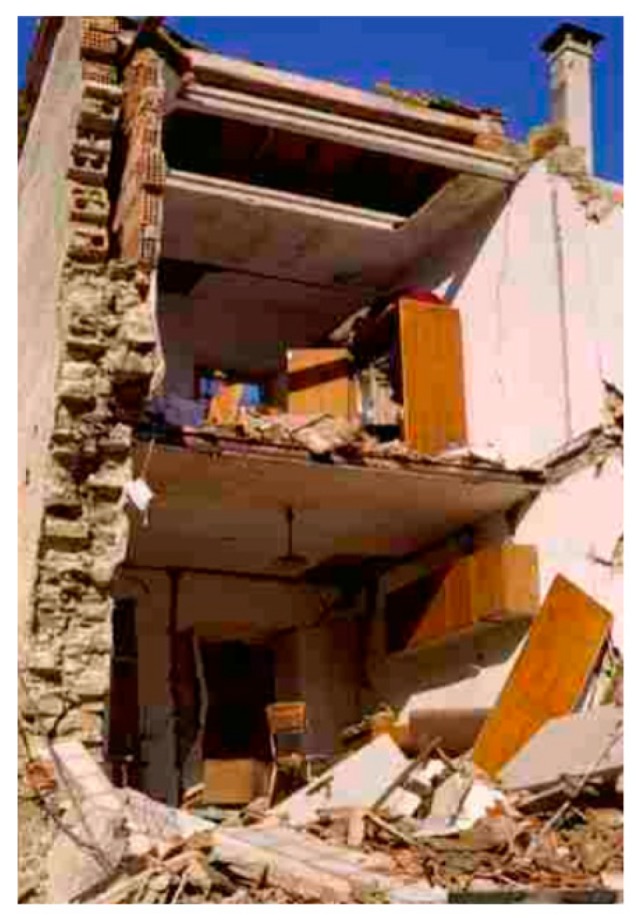
Collapse of a double-layered masonry wall [1].

Masonry is the most used material in the historical buildings of the European architectural heritage. The mechanical properties of these structures are often low, due to both the texture of the masonry and the poor quality of the mortar. In particular, masonry walls are often made up of two vertical layers (Figure 1), without any transversal links between them [1,2]. This wall geometry can produce instability problems of the external layer under the combined action of vertical and out-of-plane loads. Furthermore, masonry buildings usually have wooden horizontal floors without any effective floor-to-walls connections. This increases the actual slenderness of each wall layer when the out-of-plane actions load the masonry walls, in addition to the in-plane compressive and shear forces. Moreover, when a single layer forms the masonry wall, very often the wall texture is irregular. In the south-center Apennine area, for example, traditional masonry is made of calcareous stones of different size, almost knobble or rough-shaped, sometimes chaotically arranged, connected by low quality lime mortar [3]. As a final introductory remark, it is worth noting that, both in double and single layer walls, some parts of the same wall are often made of different materials, making the wall non homogeneous [4] (Figure 1).

The previous peculiarities make European historical structures particularly vulnerable to extreme loads, such as those related to the effects of impact [5,6,7,8,9], blast [10,11,12,13,14], fire [15,16,17,18,19], and earthquake [20,21] actions. In particular, they are extremely vulnerable to the earthquakes even for low-medium intensity, as some recent inestimable damages in Mediterranean regions testify. Therefore, strengthening of masonry structures is a topic of primary importance in Europe.

Recent studies in earthquake engineering are oriented to the development, validation, and application of techniques to assess the seismic vulnerability of existing masonry buildings [22]. As far as the seismic risk in Italy is concerned, in 2011 Rota et al. [23] plotted typological seismic risk maps for the entire national territory, where the typological seismic risk is the convolution of vulnerability and hazard for a building belonging to a given typology.

To build up the maps of the typological seismic risk, Rota et al. used data collected during post-earthquake surveys, after the earthquakes of Irpinia (1980), Abruzzo (1984), Umbria-Marche (1997), Pollino (1998), and Molise (2002), on more than 91,000 buildings. Subsequently, they assessed the vulnerability by adopting a damage scale similar to that defined in the European Macro-seismic Scale: five damage levels (from DS1 to DS5) in addition to the no damage case (DS0) make up the damage scale, as shown in Table 1.

**Figure 2:**
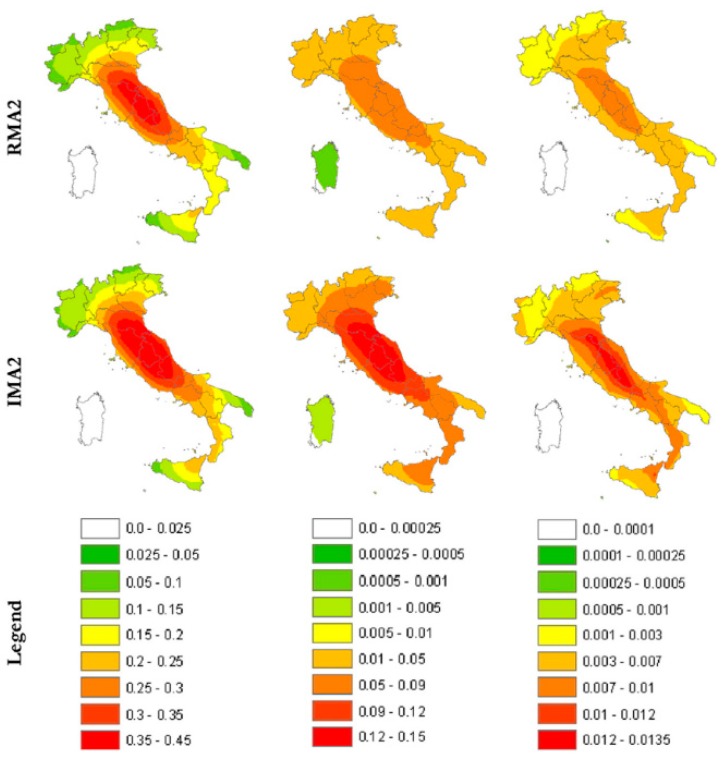
Italian annual probability of exceeding DS1, DS3, and DS5 (in the columns) for the building typologies RMA2 and IMA2 defined in Table 2 (in the rows) [23].

Rota et al. computed the damage level for 23 building typologies. For the purposes of this paper, however, the authors will consider only the building typologies collected in Table 2. As shown in Figure 2, the irregular layout is a serious factor of risk, since it increases the vulnerability of masonry structures further. Note that the color scale in Figure 2 is different for each damage level, but is the same for a given damage level, therefore allowing direct comparisons between typologies, once established the damage level of comparison.

**Figure 3:**
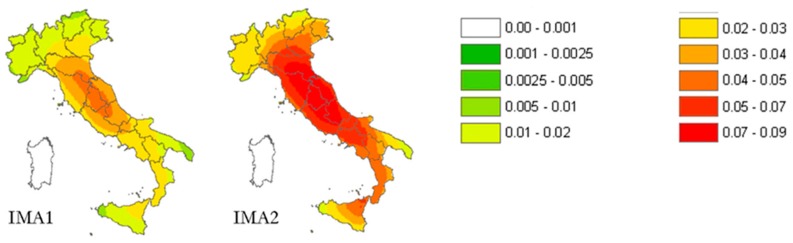
Italian risk maps of the annual damage factor for masonry buildings with (IMA1) and without (IMA2) tie rods and/or tie beams [23].

Figure 3 shows the effect of connections on the annual damage factor for low-rise masonry buildings with irregular layout (IMA1 and IMA2, as defined in Table 2): the annual probability of losing the building is significantly higher when there are neither tie rods nor tie beams connecting the various structural elements of the building. In fact, when computing the average annual damage factor over the Italian territory for all the 23 building national typologies, the typologies with the highest national average annual damage factor are exactly those of the type IMA2, the irregular layout masonry buildings with flexible floors and without any tie rods and/or tie beams. This means that ensuring a box-type behavior with good structural connections is of primary importance in retrofitting masonry buildings, particularly when the layout is irregular.

In the following Sections, the authors will compare some of the latest masonry strengthening techniques, with particular focus on the ability to restore or improve the box-type behavior.

## 2. State of the Art on Retrofitting Techniques for Masonry Structures

When dealing with the structural performance of masonry structures, the two major concerns are compressive and shear overloads, both under static and dynamic loads. Nowadays, there is a large variety of available techniques and materials for interventions on historical masonry constructions. Among them, two main techniques are distinguished [22]: rehabilitation (or restoration) and retrofitting. Rehabilitation uses materials of characteristics similar to the original ones and applies the same construction techniques, in order to correct the local damage of structural elements. In general, the objective of these works is to preserve the building in good condition and in its original state, mainly to withstand the vertical loading generated by self-weight (dead load). Conversely, structural retrofitting intends to use modern techniques and advanced materials to improve the seismic performance of the building, by increasing its ultimate lateral load capacity (strength), ductility, and energy dissipation.

There are many techniques, in the literature, proposed in the past to increase the masonry strength for both compression and shear overloads or to restore the masonry performance after damage. Most of these techniques were derived from experiences on the use of FRP (Fiber Reinforced Polymer) to enhance the load-bearing capacity of concrete structures. This family of reinforcement techniques allows increasing the local strength of the single structural element greatly, but, in most cases, does not have a significant impact on the overall performance of the structure, since attaining satisfactory connections between all the structural elements of the same structure is not easy at all. Consequently, increasing the stiffness of the weakest structural element generally results in an increased vulnerability of the adjacent ones or the structural connections. This latter case compromises the box-type behavior of the building.

Moreover, the solutions adopted in historical masonry structures are usually subjected to some limitations and recommendations from heritage conservation organizations and statutory bodies, like the requirement of not changing the aesthetical and architectural value, often remarkable, which marks the border between a structure, so to speak, simply old and one of historical interest. In general, in the case of retrofits for the seismic protection of cultural heritage, it is essential to take into account the compatibility, durability, and reversibility (removability) of the intervention. Since FRP reinforcements are not always able to guarantee a conservative solution and the weakness of masonry connections is higher than the weakness of concrete ones, it is necessary to promote the use of new materials, capable of satisfying both safety and conservation.

In the following Sections, the authors will discuss the effectiveness of some innovative techniques of retrofitting, with particular focus on the techniques of active reinforcement.

### 2.1. Active and Passive Strengthening

Every current method of structural reinforcement falls into one of the two fundamental strengthening approaches, either passive or active reinforcement. The difference between these two major families of reinforcement techniques consists of how the structural retrofitting takes place: the strengthening elements of a passive reinforcement receive loads only from the structural element, when it deforms further, whereas the strengthening elements of an active reinforcement have a pre-load that counteracts the deformation of the structural element from the moment of installation.

For example, in the case of compressed, passively confined structural elements, the confinement pressure depends on the incremental lateral expansion of the reinforced element, generated by the axial load applied after retrofitting, due to the Poisson effect [24]. Therefore, if the incremental axial load is nonexistent or relatively small, the confining pressure is negligible and the external confining material does not have any effect on the load-deformation behavior of the structural element. Furthermore, in order to take full advantage of the confinement material, the structural element must have already undergone at least some type of damage [25]. Lastly, the stiffer the structural element, the less effective the passive confinement.

With the active confinement method, on the contrary, the confinement material provides the confinement pressure to the structural element, independently of the lateral strain. This means that the confinement pressure depends only on the material used and its stress of post- or pre-loading. The main advantage of this technique is that there is no need for damage to take full advantage of the confinement material.

### 2.2. Some Recent Active Retrofitting Techniques For Masonry Buildings

#### 2.2.1. Punctual Retrofitting Techniques

The shape memory effect of SMA (Shape Memory Alloy) materials seems to be an innovative suitable solution for the active strengthening of masonry structures [26]. In fact, it is possible to use SMA materials together with FRP wrapping, which provides a passive strengthening, to activate confinement in masonry columns [27]. Nevertheless, being an improvement of FRP applications, this technique inherits from FRPs the peculiarity of being a technique for local strengthening. Thus, its effectiveness in masonry buildings seriously depends on the quality of the structural connections.

The strengthening category of “horizontal and vertical ties”—one of the four categories of strengthening techniques considered in Italian seismic codes [28,29]—is particularly suitable in the cases of not effective connections between walls or between walls and floors. In fact, the use of metal ties in structures made of brick masonry dates back to load-bearing masonry walls in the 1850’s [30]. Specifically, the first use of ties in the walls of brick masonry constructions took place in England, by using wrought iron ties in brick masonry cavity walls. Since then, the addition of different types of metal bars has become a common practice in interventions on old constructions.

In their early applications, metal ties were horizontal bars, used to eliminate the horizontal thrust of arches, vaults, and roofs, while the use of vertical tie-bars for reinforcement purposes became a custom only later. Both horizontal and vertical metal tie-bars are suitable to provide a better connection between structural elements at the floor level, ensuring a box-type behavior of the entire structure, but they act in different ways on the structure. In fact, while the horizontal tie-bars allow avoiding all the out-of-plane turnover mechanisms of masonry walls, the vertical tie-bars are effective in avoiding every in-plane rotation of masonry elements. In both cases, it is fundamental to protect the metal elements against corrosion by means of a suitable covering or galvanization zinc plating or, in extreme cases, using stainless steel elements. Another disadvantage of this retrofitting system is the heavy weight of the metal bars.

**Figure 4:**
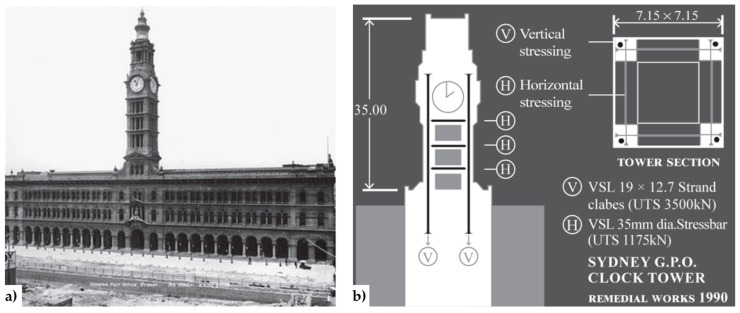
The General Post Office (GPO) Tower (Sydney, Australia): (**a**) external overview of the GPO façade; (**b**) strengthening scheme with the internal horizontal and vertical tie-bars [22].

**Figure 5:**
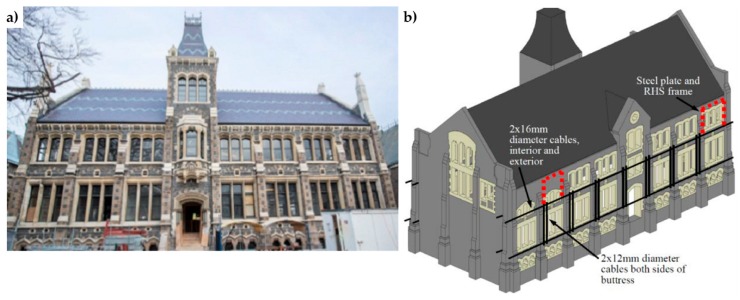
Christchurch Arts Centre, Chemistry building (New Zealand): (**a**) external overview of the façade; (**b**) horizontal and vertical cables for external post-tensioning, paired with companion horizontal tendons running parallel on the inside of the wall in order to enhance a frame-type action of building response.

Depending on the aesthetical and architectural characteristics to preserve, it is preferable to install the tie-bars inside, rather than outside the masonry elements. In existing structures, the housing of internal tie-bars is made by drilling the walls (Figure 4 [22]) while, in new buildings, it is made by anchoring one end of a high-tensile steel rod, applying any additional corrosion protection and building the brickwork section around it [31]. One of the main advantages of internal arrangements is that they protect steel against corrosion. In the case of external arrangements, the tie-bars run near the walls or in grooves cut on the wall surface. When the vertical tie-bars are external and unbounded, they are discretely located at the wall corners or next to buttresses (Figure 5) such that architectural impacts can be minimized [32].

Both for the inside and the outside arrangement, the anchorage is guaranteed by metal or concrete end plates that also allow the pre-stressing of the bars: in the first case (inside arrangement), post-tensioning can either be bonded when tendons are fully restrained, by grouting the cavity, or left unbounded by leaving cavities unfilled.

Post-tensioning of masonry by means of vertical tie-bars offers the possibility to introduce any desired level of axial load in a wall to enhance strength, performance, and durability of masonry structures [32,33,34,35,36]. In particular, the level of seismic improvement strongly depends on the level of pre-stressing force [37,38]. In fact, the compressive force provided by the vertical tendons enhances the strength, cracking behavior, and ductility of the masonry walls, as well as having a restoring or self-centering effect, by reducing residual deformations after loading [39,40,41,42]. Moreover, the pre-stressing helps avoid brittle tensile failure modes of masonry walls and offers major advantages for the connection of vertical and horizontal members in precast construction [43].

**Figure 6:**
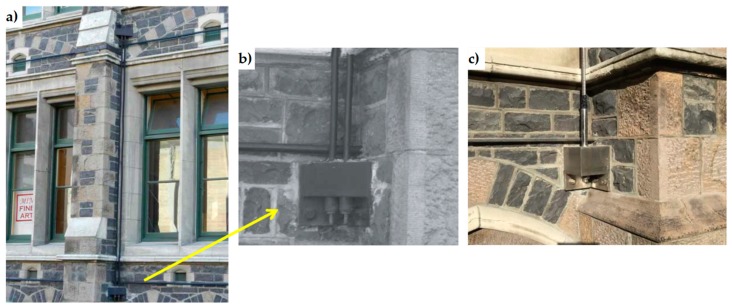
Christchurch Arts Centre, Chemistry building: (**a**) external vertical cables connected to the structure through junction boxes, to increase the compression caused by gravity loads and ensure that the wall stays in overall compression during shaking; (**b**) retrofit dating back to 1984, with pairs of external unbonded tendons; (**c**) post-earthquake retrofit, with a stainless steel cable.

During the 2010/2011 Canterbury earthquake sequence, the actual effectiveness of post-tensioning unreinforced masonry (URM) was demonstrated by the performances of the Chemistry (Figure 5) and College Hall buildings—two stone masonry buildings within The Arts Centre of Christchurch (New Zealand)—which received post-tensioned seismic retrofits in 1984 [44]. Although the retrofits were subject to considerable budgetary constraints and both pre-stress losses and corrosion had decreased the efficiency of the retrofit system after 26 operating years, the post-tensioning succeeded in improving the in-plane and out-of-plane wall strength significantly and limiting residual wall displacements. Consequently, the original post-tensioning system was renewed and reinstated after the seismic sequence, this time using steel cables (Figure 6) in order to avoid corrosion phenomena.

It is worth mentioning that even the idea of post-tensioning unreinforced masonry dates back to the XIX century and found some of its early applications in England: the oldest known post-tensioning method in England is the one utilized in 1825 to dig tunnels under the River Thames. In the same period, the post-tensioning of masonry found application also in Italy, in the Roman Coliseum, to connect the internal walls, perpendicularly located, to the external ring, in order to protect them against out-of-plane loading that could cause overturning [45,46].

The weak-point of a post-tensioning method with metal bars is that there is no control or monitoring of the pre-stressing force, which changes throughout the years by temperature, corrosion, and relaxation due to deformation of masonry (creep).

**Figure 7:**
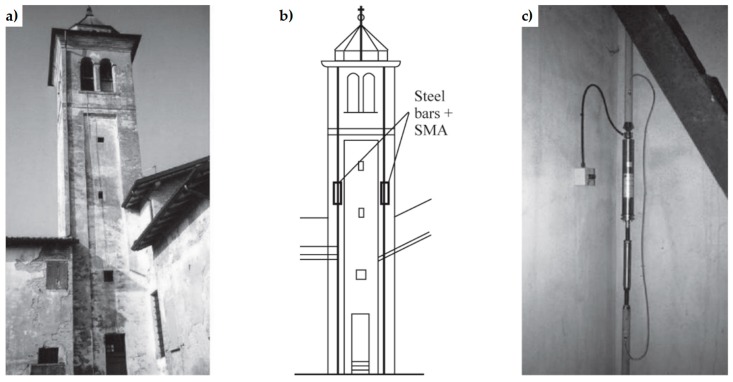
The bell tower of the church of San Giorgio in Trignano (Italy): (**a**) external view; (**b**) strengthening scheme; (**c**) detail of the coupling between Shape Memory Alloy (SMA) and a vertical steel tendon [22].

An attempt to keep the applied force constant is represented by the combined device of the church of San Giorgio in Trignano, Italy (Figure 7), where SMA and vertical steel tendons were used together to increase bending and shear resistance.

The difficulties to generate a good connection between bars and the excessive concentration of stresses induced by the anchorage to the masonry could lead to crushing. Also for these reasons, past intervention techniques in ancient masonry towers found application more as local strengthening of certain vulnerable structural parts than for a real improvement of the global behavior of the structure against earthquakes.

In [47], Darbhanzi et al. provide one of the few investigations on the effectiveness of using vertical steel strips to improve seismic behavior of unreinforced masonry walls.

#### 2.2.2. Continuous Retrofitting Techniques

**Figure 8:**
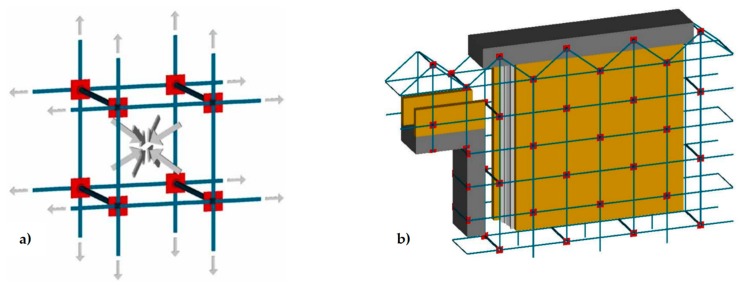
The Active Confinement of Masonry (CAM) system: (**a**) desired stress-transfer scheme from the ribbons of the rectangular arrangement to the confined masonry (hydrostatic compression); (**b**) connections between a double layer vertical wall, the upper reinforced concrete (RC) kerb and a door [1].

In 1999, Dolce and Marnetto patented the CAM (Active Confinement of Masonry) system, a reinforcement technique that overcomes the logic of the building as a juxtaposition of single structural elements, since it faces the retrofitting of masonry structures as a whole [48]. The key-idea that allows this change of viewpoint is the use of a continuous three-dimensional system of pre-tensioned ties, able to “pack” the masonry structure, thus providing an advantageous state of tri-axial compression. Actually, the main target of the CAM system is to improve the strength capabilities of masonry by adding a hydrostatic state of stress to the operational loads (Figure 8a). In Section 3.2 the authors will discuss whether the CAM system actually allows achieving this goal or not.

The CAM system does not use bars to create ties: it consists of steel ribbons that form horizontal and vertical loops, passing through transverse holes. The flexibility of the system allows rectangular (Figure 8a), rhombic, triangular, and irregular arrangements of the mesh. Moreover, the use of two staggered meshes, with the holes arranged in quincunxes as in Figure 8b, minimizes the number of holes. The ribbons (1–4 per loop) are clamped with a special tool that is able to apply a pre-stressing force, thus providing an active confinement to the masonry wall (Figure 8a). Therefore, the CAM ribbons strengthen the masonry in the same way as the metallic straps strengthen the packages in heavy applications. Because of this analogy, the authors will call the tensioned ribbons of the CAM system “the straps”.

The pre-stressed steel ribbons behave like tie rods opposing to both deformation and disconnection of the building elements [2]. In particular, since the straps form both horizontal and vertical closed loops, the CAM ribbons replicate the reinforcement scheme with horizontal and vertical ties. Nevertheless, the overall behavior of the CAM system is very far from that of traditional pre-tensioned horizontal and vertical ties, as the loop-shaped CAM ribbons bring several benefits [49]:

- It is no longer necessary to anchor the ties into the masonry, because the ribbons close on themselves. This eliminates the problem of the excessive concentrations of stresses induced by the anchorages.
- The straps are made of stainless steel. This avoids the typical corrosion problems of tie rods [50], which need of a suitable covering or galvanization zinc plating.
- The cross-section of the straps is very small. This allows a moderate increase in the total weight of the structure, useful to not increase the attraction of seismic forces too much.
- Each strap is a bi-dimensional device. This allows the ribbons to provide in-plane and transversal post-compression at the same time.
- The steel ribbons continue to wrap masonry even after masonry crushing. This is of fundamental importance for safeguarding life, as people do not risk that some part of the structure hits them, due to building collapse.

The active confinement provided by the straps compacts the masonry wall and, if the wall is double layered (Figure 8b), improves the transversal links between the vertical layers. It is worth noting that also masonry jacketing—made of shotcrete and light steel net reinforcement—is suitable for connecting the vertical layers of a double-layered wall. Nevertheless, jacketing is a passive strengthening and, as such, suffers all the typical drawbacks of a passive reinforcement (discussed in Section 2.1). Moreover, it is preferable to avoid the use of concrete in old masonry buildings, to eliminate deformation incompatibilities between masonry and concrete and increases in mass and/or stiffness that enhance the attraction of seismic forces [51].

**Figure 9:**
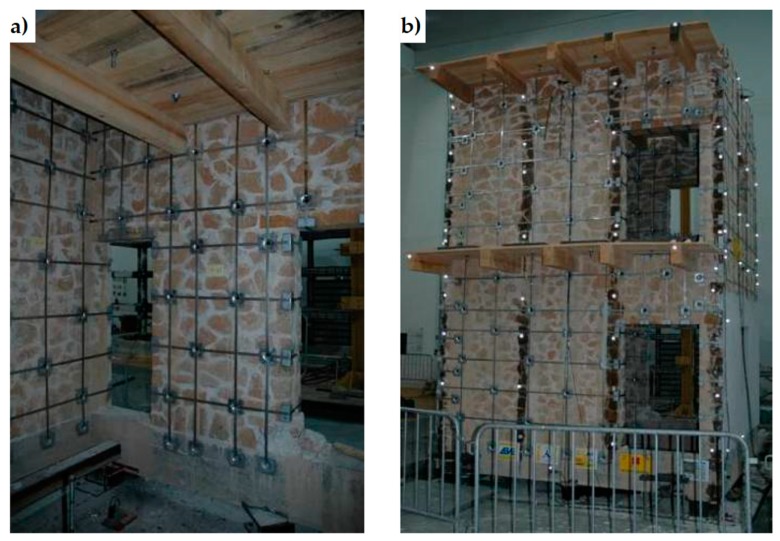
An example of reinforcement with the CAM system: 2:3-scale model for testing on a shaking table [48]: (**a**) internal view; (**b**) external view.

By running all along the masonry walls, both horizontally and vertically (Figure 9), the CAM system links together all the structural elements, thus establishing new wall-to-wall and floor-to-wall links and improving the existing connections between different structural elements, such as orthogonal walls, masonry and top kerb (Figure 8b), and masonry and wooden beams. This gives rise to a box-type behavior, if lacking, and prevents out-of-plane mechanisms. In the particular case of the scaled structure shown in Figure 9, the model was tested by applying an increasing Normalized Peak ground Acceleration (NPA) up to 1.12 g, showing only minor damages, while an unreinforced model with the same geometry collapsed for NPA = 0.31 g [48,52].

The CAM system is quickly applicable and highly reversible. The application of the system to existing structures requires the execution of small transverse holes, for the straps to pass through the wall. Since the total thickness of the straps is of the order of 6–8 mm, it is possible to contain the confining device within the normal plaster. This allows covering both the holes and the straps with mortar and plaster, hiding the reinforcement system under the surface.

**Figure 10:**
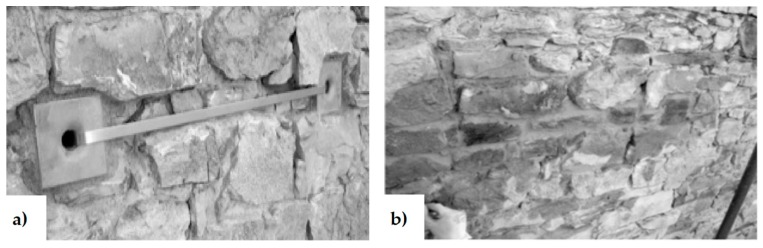
How to hide the CAM ribbons to meet the aesthetic requirements: (**a**) arrangement in slit of a steel ribbon and its protective steel plates; (**b**) restoring of the cover stone material.

**Figure 11:**
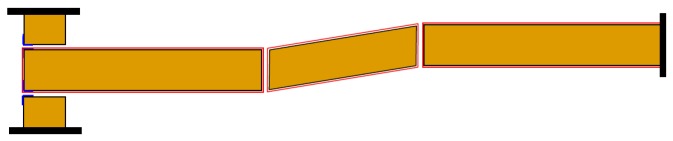
How to drill an irregular wall to allow the CAM ribbons to adapt to irregularities [1].

In those cases where the aesthetic requirements do not allow covering the surface of the wall with mortar and plaster, it is possible to house the ribbons in grooves—obtained by removing a superficial thin layer of the masonry (Figure 10a)—and restore the removed material after having clamped the straps (Figure 10b). Therefore, the technique is also minimally invasive from an aesthetic point of view. This makes the CAM system suitable for strengthening also masonry structures of historical interest. Lastly, the CAM loops can follow any irregular horizontal or vertical morphology of the wall (Figure 11). This feature and the possibility to use free-form meshes for the CAM net eliminate any difficulty to apply the CAM system in ornamented or complex-shaped walls.

**Figure 12:**
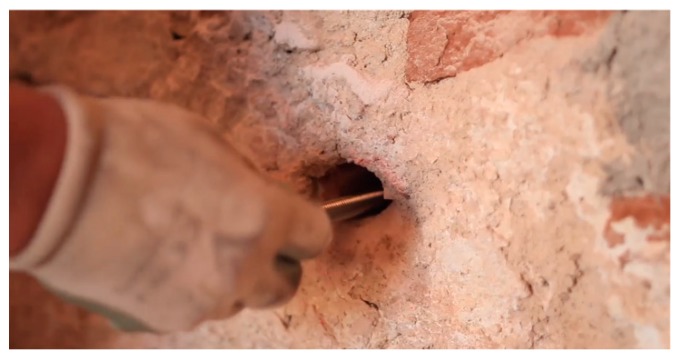
Housing of a threaded bar in a drilled hole [54].

**Figure 13:**
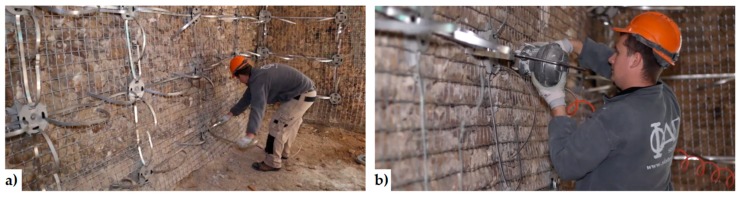
How to tie a masonry wall with the Φ system: (**a**) housing of the ribbons on the internal face of the wall; (**b**) clamping of ribbons [54].

**Figure 14:**
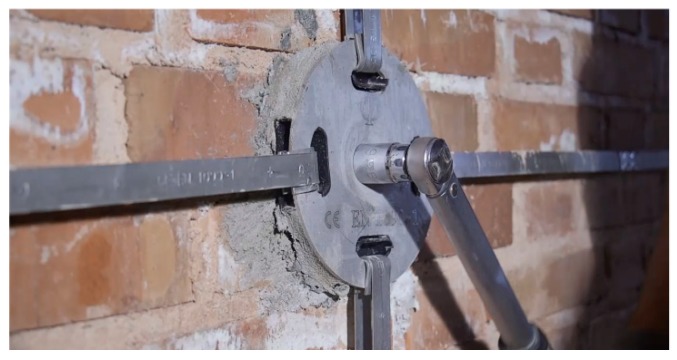
Tightening of a threaded bar [54].

Another continuous retrofitting system with stainless steel ribbons is the Φ system [53]. This latter retrofitting system is three-dimensional as the CAM system, but the ribbons do not pass through the thickness of the wall: some threaded bars make the transverse links (Figure 12), while the horizontal and vertical steel ribbons form flat loops on the internal and external faces of the wall (Figure 13). Once the ribbons have been clamped (Figure 13), the threaded bars are tightened with a torque wrench (Figure 14), providing a transverse compression to the wall. The overall behavior after retrofitting is elastic-perfectly plastic.

Due to the small thickness of the Φ ribbons, it is possible to house them in grooves as for the CAM system. Furthermore, even the Φ system can easily follow the irregularities of the wall.

Since the stress of the Φ ribbons can differ from the stress of the threaded bars, the in-plane post-compression stress can differ from the out-of-plane (transverse) post-compression stress. Actually, the post-compression stress may differ even along the two directions of the midplane: as the post-tensioned vertical ties are applicable only if the masonry is capable to bear a vertical overload, it is convenient to stress the horizontal ribbons only, leaving not loaded, or slightly loaded, the vertical ribbons. Anyway, in most real applications the stresses in both the vertical and the horizontal ribbons are close to zero. This means that the Φ system modifies the stress field of the masonry wall only along the transverse direction, leaving unchanged the compression stresses along the horizontal and vertical directions.

As the patents of both continuous retrofitting systems are relatively recent, the related strengthening mechanisms are, in part, still unknown. In particular, in the authors’ opinion the stress-transfer scheme assumed for the CAM system (Figure 8a) is questionable and hardly complying with the actual additional state of stress provided by the CAM system to a masonry wall. Since the scheme in Figure 8a is the core idea that also inspired the design criteria of the CAM system, this could mean that the design criteria of the CAM system are incorrect.

With the aim of providing a contribution to rise discussions and improvements on the design criteria of both continuous retrofitting systems, in the following Sections the authors will focus on the actual strengthening mechanisms of the CAM and Φ systems. In the spirit of a first analysis, the authors will investigate the stress-transfer mechanisms under static conditions, making use of a stress analysis in the Mohr/Coulomb plane. In Section 4, the authors will also introduce a critical analysis of the design criteria of the CAM system, identifying the most relevant shortcomings.

## 3. An in-Depth Study of the Three-Dimensional Continuous Systems: The Actual Strengthening Mechanisms

The purpose of this Section is to investigate the actual benefits of the two continuous three-dimensional strengthening systems: the CAM system and the Φ system. The comparison will allows understanding which retrofitting system is more performing.

### 3.1. The Φ System

By starting the analysis on the continuous three-dimensional strengthening systems from the Φ system, the first question to ask is what value of the transverse stress optimizes the performances of a masonry wall. Indeed, the answer to this question is by no means trivial.

**Figure 15:**
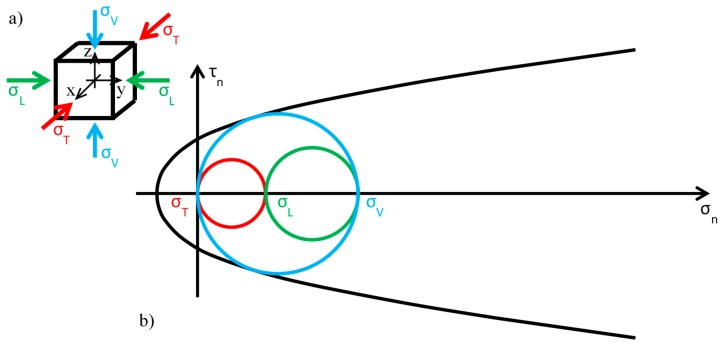
Stress analysis in the Mohr/Coulomb plane: (**a**) stresses acting on the infinitesimal volume of the masonry wall; (**b**) limit condition in the plane of Mohr/Coulomb before the application of the retrofitting system.

For the sake of simplicity, the authors assumed that the stress in the ribbons is equal to zero and the transverse stress is constant, applied continuously to the wall by the retrofitting system. In these assumptions, each infinitesimal volume of the masonry wall is stressed as shown in Figure 15a, where $\sigma_{T}$ is the transverse stress (out-of-plane stress provided by the retrofitting system), $\sigma_{V}$ is the vertical stress (due to self-weight), and $\sigma_{L}$ is the lateral stress (function of $\sigma_{V}$ by means of Poisson’s ratio).

Before the retrofitting system is applied, there are no constraints along the transverse direction of the wall and the out-of-plane stress is equal to zero:

(1) $$\sigma_{T}=0$$

Figure 15b shows the static limit condition in the plane of Mohr/Coulomb for $\sigma_{T}=0$, with the limit surface approximated by making use of the parabolic domain of Leon:

(2) $$\tau_{n}^{2}=\frac{c}{f_{c}}\left(\frac{f_{tb}}{f_{c}}+\sigma_{n}\right)$$

as usually done for masonry [55] and, more generally, for brittle materials [56,57,58,59,60,61]. In Equation (2), $\tau_{n}$ is the total shear stress, c the cohesion, $f_{c}$ the compressive strength, $f_{tb}$ the tensile strength, and $\sigma_{n}$ the normal stress. Moreover, in Figure 15b the authors assumed that the stresses of compression are positive.

Since the greatest circle of Mohr is associated with the z/x plane of Figure 15a (the blue circle in Figure 15b), the crisis occurs in a plane parallel to the *y* axis (sliding in the thickness of the wall), when the self-weight reaches a limit value depending on the shape of the parabolic domain.

**Figure 16:**
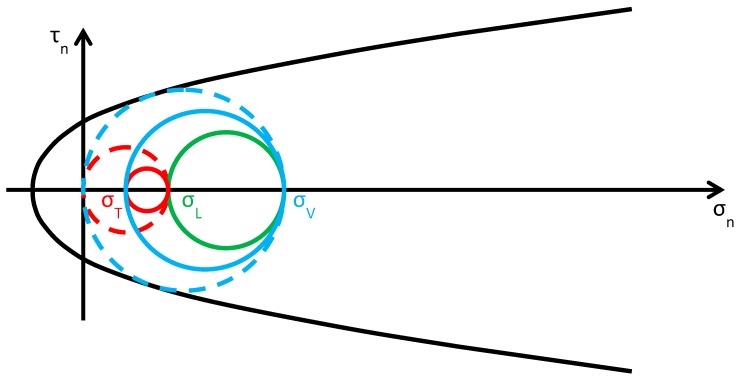
Stress analysis in the plane of Mohr/Coulomb after retrofitting, for $0<\sigma_{T}\leq \sigma_{L}$ (Mohr’s circles before retrofitting in dashed lines, for comparison).

**Figure 17:**
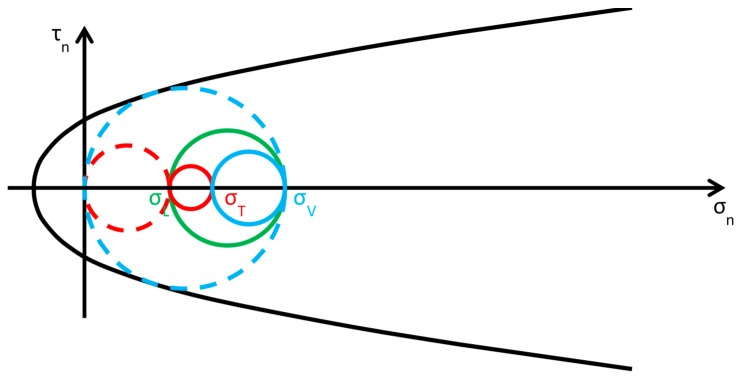
Stress analysis in the plane of Mohr/Coulomb after retrofitting, for $\sigma_{L}<\sigma_{T}\leq \sigma_{V}$ (Mohr’s circles before retrofitting in dashed lines, for comparison).

**Figure 18:**
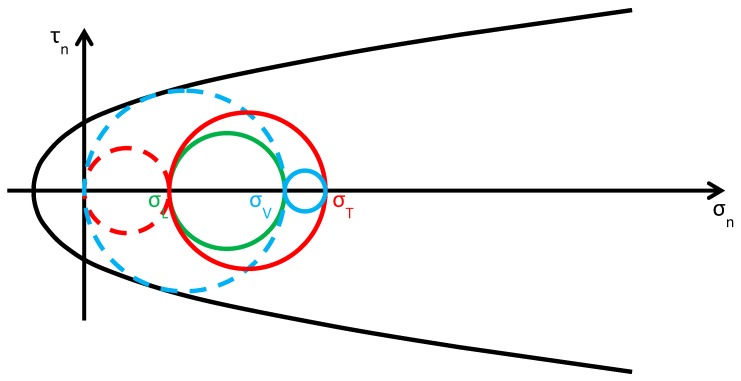
Stress analysis in the plane of Mohr/Coulomb after retrofitting, for $\sigma_{T}>\sigma_{V}$ (Mohr’s circles before retrofitting in dashed lines, for comparison).

**Figure 19:**
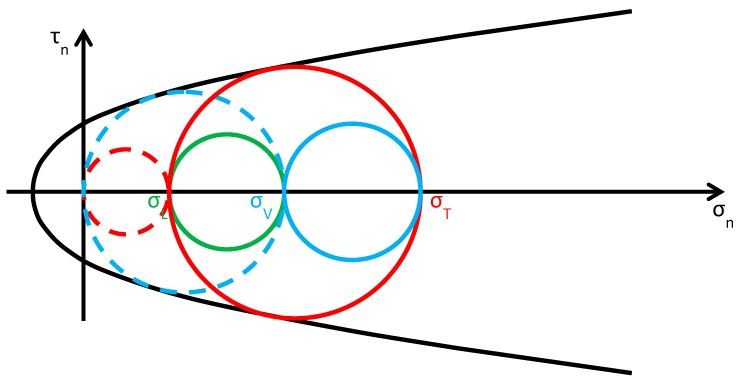
First limit condition after the application of the retrofitting system (Mohr’s circles before retrofitting in dashed lines, for comparison).

As discussed in Section 2.2, usually the Φ system does not modify the lateral and vertical stresses ($\sigma_{L}$ and $\sigma_{V}$) significantly, while it provides an additional out-of-plane stress ($\sigma_{T}$). Consequently, the radius and the position of the circle of Mohr associated with the y/z plane (the green circle in Figure 15b) do not change after retrofitting, while the radii and the positions of the remaining two circles change in function of the final value assumed by $\sigma_{T}$. By increasing $\sigma_{T}$ monotonically, starting from the initial value $\sigma_{T}=0$, the fields of behavior are three (the authors have assumed that the initial condition is a limit condition):

- $0<\sigma_{T}\leq \sigma_{L}$ (Figure 16): the greatest circle is associated with the z/x plane (blue circle). Both the red and blue circles become smaller and move away from the limit surface. This increases the minimum distance between the greatest circle and the limit surface, which provides a measure of the safety factor. Thus, the higher the value of $\sigma_{T}$ in this interval, the higher the safety factor. In other words, the retrofitting intervention is effective in this field. More precisely, it is all the more effective the higher the out-of-plane post-compression. At the end of the interval, when $\sigma_{T}=\sigma_{L}$, the red circle degenerates into a point and the blue circle superimposes onto the green circle.
- $\sigma_{L}<\sigma_{T}\leq \sigma_{V}$ (Figure 17): the greatest circle is associated with the y/z plane (green circle). When the out-of-plane compression, $\sigma_{T}$, increases from the value $\sigma_{L}$ to the value $\sigma_{V}$ (in absolute value), the radius of the red circle increases while the radius of the blue circle decreases. It could seem that the safety factor does not change in this interval: since the radius of the greatest (green) circle does not modify, the safety factor does not seem to depend on the value of $\sigma_{T}$. In fact, the discussion about the safety factor is a bit more complex. As a matter of fact, retrofitting the masonry wall modifies the overall behavior of the wall, that is, modifies the limit surface, all the more greater as the stress of the threaded bars increases. The new limit surface is a combination of the two limit surfaces of masonry and steel. Thus, it seems reasonable that the new limit surface is wider and flatter than the limit surface in Figure 17. In conclusion, if computed as the minimum distance between the greatest circle and the combined limit surface, the safety factor slightly increases even in this interval. At the end of the interval, when $\sigma_{T}=\sigma_{V}$, the red circle superimposes onto the green circle and the blue circle degenerates into a point.
- $\sigma_{T}>\sigma_{V}$ (Figure 18): the greatest circle is associated with the x/y plane (red circle). Both the red and blue circles become greater. In particular, the red circle grows closer to the limit surface of masonry. This decreases the minimum distance between the greatest circle and the masonry limit surface. The minimum distance between the greatest circle and the combined limit surface also decreases, but slower than the previous one. In conclusion, in the third interval the combined safety factor decreases. Moreover, there are two limit values of $\sigma_{T}$: the first limit value of $\sigma_{T}$ makes the red circle tangent to the masonry limit surface (Figure 19) and the second limit value, $\sigma_{T}=\sigma_{Tu}$, higher than the previous one (in absolute value), makes the red circle tangent to the combined limit surface. The crisis takes place for the second limit value and occurs in a plane parallel to the *z* axis. Thus, the retrofitting system modifies the crisis mechanism.

In conclusion, not all the values of out-of-plane stress are advantageous for the masonry wall and it is possible that high post-compression stresses cause a decrease in the safety factor. In particular, to avoid the collapse of the wall it is necessary not to exceed the upper limit value $\sigma_{Tu}$ of $\sigma_{T}$. The value of $\sigma_{Tu}$ depends on the shape of the combined limit surface, which takes into account both the elastic properties of masonry and the retrofitting layout. Anyway, if compared with the crisis mechanism of unreinforced masonry (sliding plane parallel to the *y* axis, as for the case in Figure 15b), the post-retrofitting crisis mechanism activated for $\sigma_{T}=\sigma_{Tu}$ is less dangerous. In fact, in the first case, the sliding plane separates the wall in an upper and a lower portion, with the upper one that falls down along the sliding plane, while, in the second case, the sliding plane is vertical and the sliding occurs along a horizontal direction (the displacement vectors are parallel to the horizontal plane). Therefore, in the second case both portions (on the right and left of the vertical sliding plane) continue to stand. Lastly, the maximum benefit in terms of safety factor occurs in the first variation interval of $\sigma_{T}$, $0<\sigma_{T}\leq \sigma_{L}$, where $\sigma_{L}$ does not assume a constant value inside the wall. In fact, since $\sigma_{L}$ depends on $\sigma_{V}$ by means of Poisson’s ratio, the higher the weight of the overlying masonry the higher the value of $\sigma_{L}$. Consequently, the Φ system achieves maximum effectiveness when applied to the walls of the lower stories, where both $\sigma_{L}$ and $\sigma_{V}$ are maximum.

### 3.2. The CAM System

**Figure 20:**
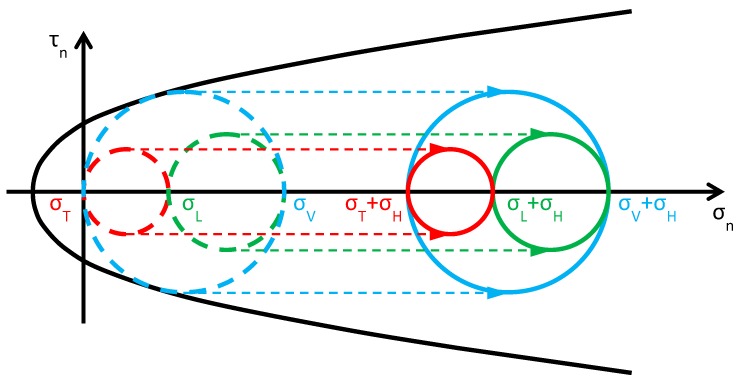
How previous papers assume that the CAM system acts on Mohr’s circles (Mohr’s circles before retrofitting in dashed lines, for comparison).

As anticipated in Section 2.2.2, the purpose of this Section is to verify whether the aim of providing a tri-axial compression state, by dividing the wall into units and packing each of them as shown in Figure 8a, is actually achieved or not by the CAM system. In particular, in Figure 8a the additional stress given by the retrofitting system is the same along each direction, that is, it is a hydrostatic state of stress. If this assumption was correct, the retrofitting would move the three circles of Mohr along the horizontal positive semi-axis for the same amount, equal to the hydrostatic stress $\sigma_{H}$, without varying their radii (Figure 20). As a result, the three circles—therefore also the biggest—would move away from the limit surface, thus increasing the safety factor.

In this case, the benefit of applying the CAM system would be theoretically unlimited, as it is possible to increase the safety factor indefinitely in the plane of Mohr/Coulomb (the only upper limit is represented by crushing [62,63]). Nevertheless, the experimental tests do not confirm the theoretical unlimited increase in load-bearing capacity. The reason for this probably lies in a basic misunderstanding concerning the model shown in Figure 8a, when extended to describe the overall behavior of retrofitted walls: the masonry units obtained by drilling the wall are not individual volumes, but interact somehow. Thus, describing the overall behavior of a retrofitted wall as the juxtaposition of free volumes in space—subjected to a hydrostatic compression like the volume of Figure 8a—is not entirely adequate.

**Figure 21:**
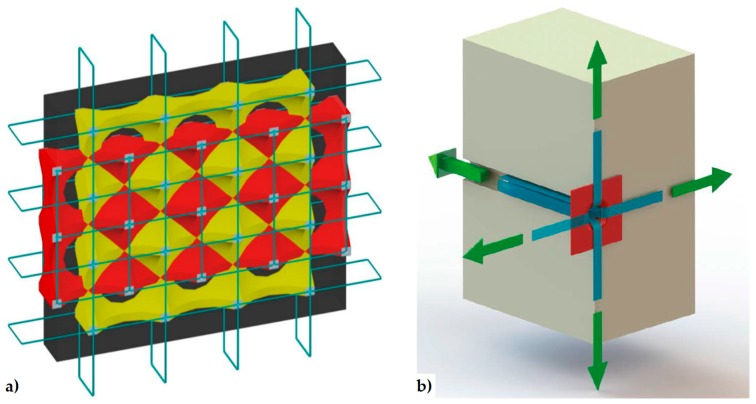
Retrofitting design of a masonry wall by the CAM system: (**a**) the internal stress-field assumed in [52,64]; (**b**) forces acting on one node of the CAM net, provided by the straps that pass through a common drilled hole [52].

**Figure 22:**
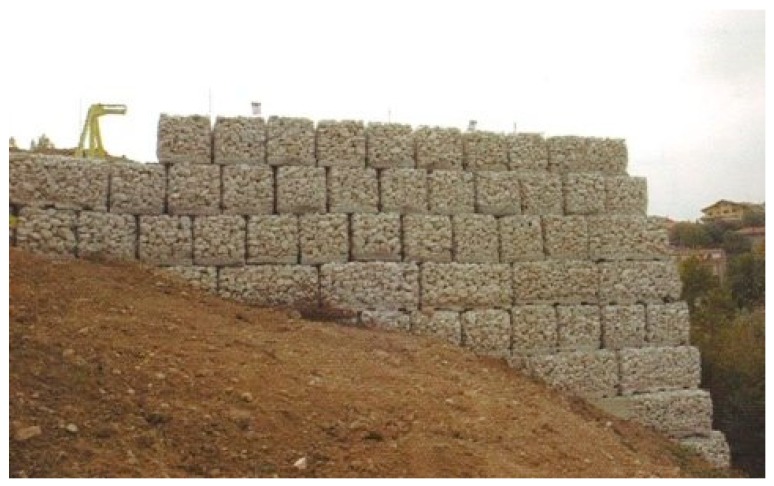
Metallic gabions for retaining walls and slope stabilization.

This misunderstanding is evident in the model adopted for the design of wall retrofitting with the CAM system (Figure 21a [52,64]). In fact, the typical stress transfer scheme of the free unit in the space of Figure 8a is juxtaposed to fill the wall volume in Figure 21a, as if the packed units do not interact in any way. In other words, the idea underlying the explicative model in Figure 21a is that the masonry units of the CAM system are placed side by side as the metallic gabions filled with stones in the retaining walls (Figure 22), with the adjunctive conditions that the “CAM gabions” compress the masonry units hydrostatically and independently of the surrounding masonry units. In reality, since the drilled holes of the CAM net are common to different masonry units (Figure 21b), each vertex of a unit is constrained by the surrounding units to an extent that depends on the position in the wall of the unit and the number of surrounding units (not necessarily three). In fact, evaluating the actual degree of constraint is not easy, because clamping and tensioning do not occur simultaneously in all straps. The order in which the straps are clamped and tensioned is very important, because relaxation and creep [65] may change the stress inside the straps and, ultimately, the constraint degree of the units.

**Figure 23:**
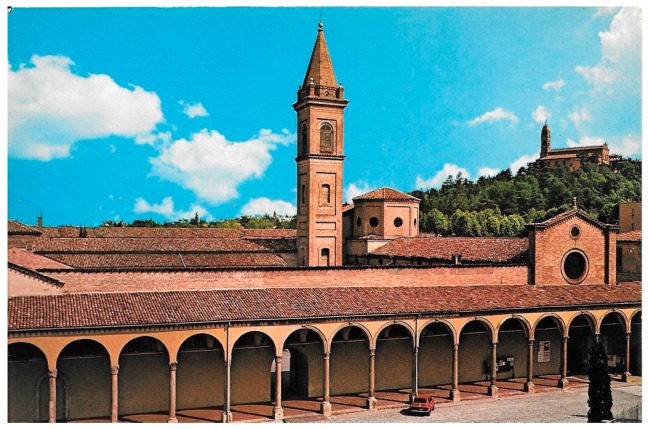
Tie rods in the portico of Chiesa di Santa Maria Annunziata, Bologna, Italy.

In the simplifying assumption that the stress is the same in all straps, the evaluation of the constraint degrees for the nodes of the CAM system is an extension to two-dimensional problems of the mono-dimensional pattern with tie rods that eliminate the horizontal thrusts (outward-directed horizontal forces) on the nodes between the frontage arches of long porticos (Figure 23). In particular, each internal node of the portico of Figure 23 receives equal and opposite thrusts from the two arches on its left and right. Therefore, the total horizontal thrust in the frontage plane for the internal nodes is equal to zero. This means that only the tie rods at the ends of the portico are actually effective, while it is possible to remove the internal tie rods (in real applications, it is common practice to also apply the internal rods to avoid local problems due to subsidence). For the same reason, the node in Figure 21b and all the internal nodes of the CAM system, being subjected to pairs of equal and opposite forces in the plane of the wall, do not receive any in-plane force from the retrofitting system. The only nodal force not balanced by an equal and opposite force is the transverse force.

**Figure 24:**
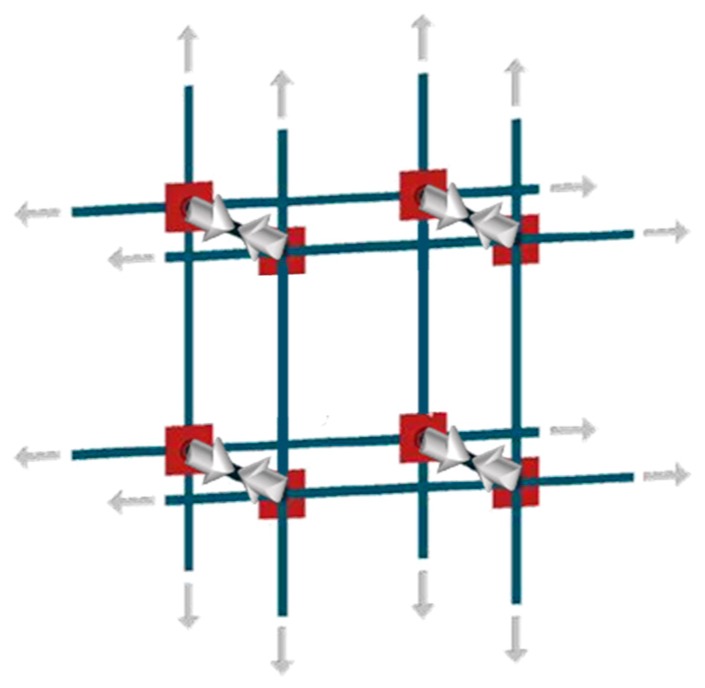
Mechanism of stress transfer in the assumption of perfectly balanced in-plane forces.

Therefore, the actual mechanism of stress-transfer from the CAM net to the masonry wall is that shown in Figure 24, which replaces Figure 8a. This means that the vertexes of the internal masonry units cannot move neither along the horizontal nor the vertical direction, but only in the transverse direction. In conclusion, the CAM system does not provide the desired strengthening mechanism, consisting of an additional hydrostatic state of stress on the masonry units.

**Figure 25:**
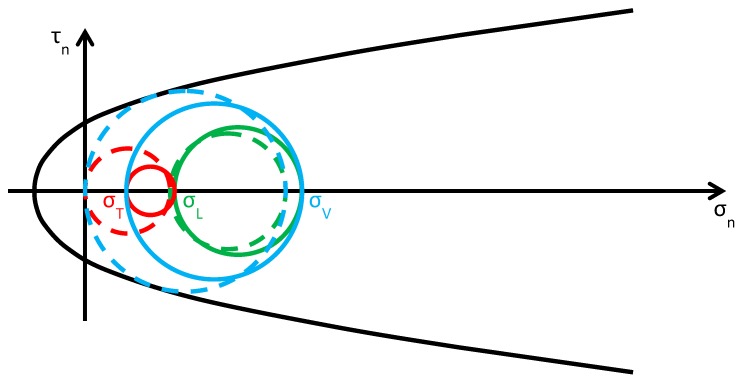
Stress analysis for $0<\sigma_{T}\leq \sigma_{L}$ (Mohr’s circles before retrofitting in dashed lines, for comparison).

**Figure 26:**
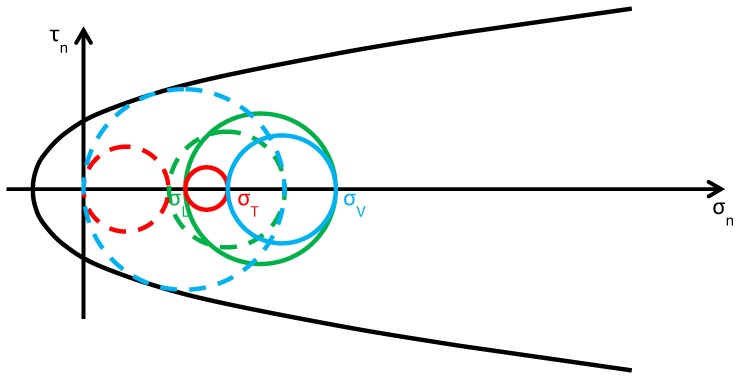
Stress analysis for $\sigma_{L}<\sigma_{T}\leq \sigma_{V}$ (Mohr’s circles before retrofitting in dashed lines, for comparison).

**Figure 27:**
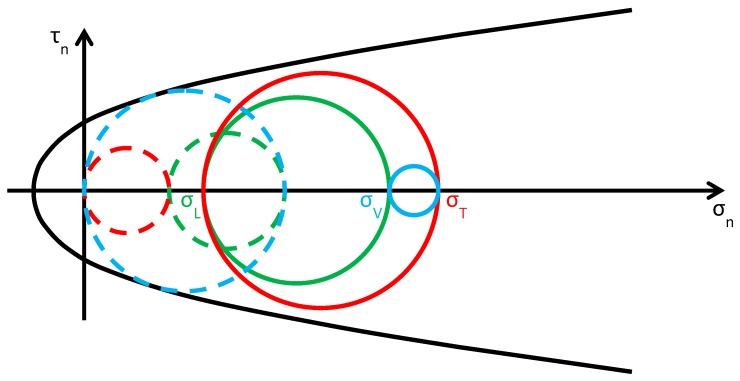
Stress analysis for $\sigma_{T}>\sigma_{V}$ (Mohr’s circles before retrofitting in dashed lines, for comparison).

Moreover, in the previous simplifying assumption that the post-tension stress is the same for all straps, the masonry units are stressed by the CAM system in same way as by the Φ system with non-tensioned ribbons and, for each given $\sigma_{T}$, the safety factor is the same for both retrofitting systems. Nevertheless, it is worth noting that this assumption is acceptable only for internal nodes of very large continuous walls and nodes of the lower stories in multi-story buildings. In fact, the constraint degree for nodes of the upper stories strongly depends on whether the building has a top kerb or not. That is, if the top kerb is absent or very deformable, the constraint to the vertical displacements is low, in particular for the nodes far from the right and left ends. Consequently, when the stress of the vertical straps increases, those nodes can move downward. This increases the total vertical stress $\sigma_{V}$ for the upper masonry units and, to a lesser extent, depending on Poisson’s ratio, even the total in-plane lateral stress $\sigma_{L}$. The modified values of $\sigma_{V}$ and $\sigma_{L}$ have a repercussion on the safety factor, which is no longer equal to the safety factor of the Φ system. In particular, for:

- $0<\sigma_{T}\leq \sigma_{L}$ (Figure 25), where $\sigma_{L}$ is the modified lateral stress, the greatest circle is associated with the z/x plane (blue circle). As $\sigma_{T}$ increases (in absolute value), even $\sigma_{V}$ increases (in absolute value), but $\Delta \sigma_{V}$, the variation of $\sigma_{V}$, is lower than $\Delta \sigma_{T}$, the variation of $\sigma_{T}$, because the constraint degree along the vertical direction is higher than the constraint degree along the transverse direction:
  (3) $$\Delta \sigma_{V}<\Delta \sigma_{T}.$$
  Due to Poisson effect, the variation of $\sigma_{V}$ ultimately causes an increase of $\sigma_{L}$, which is lower than the increase of $\sigma_{V}$ because Poisson’s ratio is lower than 1:
  (4) $$\Delta \sigma_{L}<\Delta \sigma_{V}.$$
  Both the red and blue circles become smaller, while the green circle becomes greater. The minimum distance between the largest circle and the limit surface increases, but to a lesser extent than in the case of the Φ system (for each given $\sigma_{T}$ in the interval). Thus, even for the CAM system, the higher the value of $\sigma_{T}$ in this interval, the higher the safety factor, but the post-retrofitting safety factor is lower than that achievable with the Φ system for the same $\sigma_{T}$. The CAM retrofitting is effective in this interval, all the more as higher $\sigma_{T}$ is. When $\sigma_{T}=\sigma_{L}$, the red circle degenerates into a point and the blue circle superimposes onto the green circle.
- $\sigma_{L}<\sigma_{T}\leq \sigma_{V}$ (Figure 26), where $\sigma_{L}$ and $\sigma_{V}$ are the modified lateral and vertical stresses, the greatest circle is associated with the y/z plane (green circle). As $\sigma_{T}$ increases, $\sigma_{V}$ and $\sigma_{L}$ increase as for the previous interval:
  (5) $$\Delta \sigma_{L}<\Delta \sigma_{V}<\Delta \sigma_{T}.$$
  The radii of both the red and green circles increase, while the radius of the blue circle decreases. Moreover, the center of the green circle moves along the positive semi-axis of $\sigma_{n}$. Shifting the center and increasing the radius of the green circle have opposite effects on the safety factor: the first increases the safety factor, while the second decreases the safety factor. Depending on which of the two effects prevails over the other, the safety factor can either increase or decrease. Moreover, the minimum distance between the green circle and the limit surface depends on the shape of the combined limit surface, that is, on the number of straps and their stress. In the absence of this information, it is not possible to discriminate whether the safety factor of the CAM system is higher than the safety factor of the Φ system in this interval, or not. When $\sigma_{T}=\sigma_{V}$, the red circle superimposes onto the green circle and the blue circle degenerates into a point.
- $\sigma_{T}>\sigma_{V}$ (Figure 27), where $\sigma_{V}$ is the modified vertical stress, the greatest circle is associated with the x/y plane (red circle). $\sigma_{T}$, $\sigma_{V}$, and $\sigma_{L}$ increase according to the inequalities (5). All the circles become greater, with the red circle that grows closer to the limit surface of masonry (and to the combined limit surface). This decreases the safety factor but, for each given $\sigma_{T}$, the safety factor of the CAM system is higher than that achievable with the Φ system. The crisis takes place when the red circle becomes tangent to the combined limit surface and occurs for a value of $\sigma_{T}$ that is higher than the $\sigma_{Tu}$ of the Φ system. Even for the CAM system, the retrofitting modifies the crisis mechanism, since the new sliding plane is parallel to the *z* axis.

In conclusion, the CAM system performs better than the Φ system for high values of $\sigma_{T}$, while it works worse than the Φ system for low values of $\sigma_{T}$.

## 4. A Critical Analysis of the Design Criteria for the CAM System

In Section 3.2 the authors have shown that the CAM system does not provide an additional hydrostatic state of stress to the masonry walls, disproving what the authors who treated the CAM system in the past believed. Since the idea of an additional hydrostatic state of stress is the basic assumption that inspired the development of the CAM system, this means that the design criteria of the CAM system do not match the actual mechanism of stress transfer (shown in Figure 24) and require revision. In fact, the formulas of the CAM system design manual [64] derive from the simplified model of stress transfer in Figure 21a, which does not take into account the interactions between adjacent masonry units.

**Figure 28:**
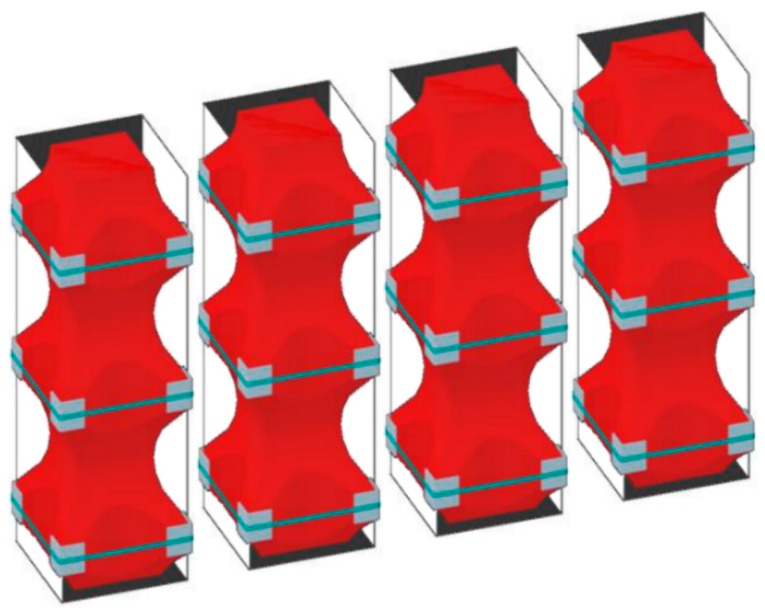
How the design criteria of the CAM system divide a masonry wall into juxtaposed confined columns to calculate the number of horizontal straps.

In particular, the design manual of Marnetto and Vari [64] distinguishes between horizontal and vertical straps, treating the horizontal straps as confinement reinforcement (like in a confined column) and the vertical straps as additional reinforcement, against out-of-plane bending. As a result, Marnetto and Vari model the masonry wall as a series of juxtaposed confined columns, which do not interact with each other (Figure 28).

**Figure 29:**
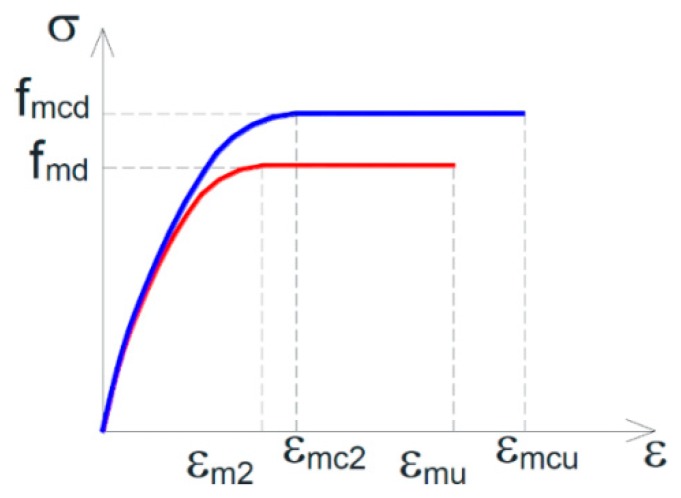
Design constitutive relationships of masonry: unreinforced masonry (URM) in red, confined masonry in blue [64].

The formula chosen in [64] to calculate the design compressive strength, $f_{mcd}$ (Figure 29), in a masonry wall that receives the confinement pressure $f_{1}$ from the horizontal straps of the CAM system is:

(6) $$f_{mcd}=f_{md}\left[1+k^{\prime }{\left(\frac{f_{1,eff}}{f_{md}}\right)}^{\alpha_{1}}\right];$$

where:

- $f_{md}$ is the design compressive strength of the unreinforced masonry (URM);
- $k^{\prime }$ is a dimensionless coefficient of strength increase, which depends on the mass density, $g_{m}$, through the relationship:
  (7) $$k^{\prime }=\alpha_{2}{\left(\frac{g_{m}}{1000}\right)}^{\alpha_{3}},$$
  with $g_{m}$ expressed in $kg/m^{3}$ and both coefficients $\alpha_{2}$ and $\alpha_{3}$ equal to 1 (in the absence of proven experimental results that justify different assumptions);
- $f_{1,eff}$ is the effective confinement pressure, that is, the confinement pressure $f_{1}$ reduced by a coefficient of efficiency, $k_{eff}\leq 1$, defined as the ratio between the effectively confined volume of the masonry wall, $V_{c,eff}$, and the volume of the masonry wall, $V_{m}$:
  (8) $$f_{1,eff}=k_{eff}\cdot f_{1},$$
  (9) $$k_{eff}=\frac{V_{c,eff}}{V_{m}};$$
- $\alpha_{1}$, in the absence of proven experimental results, is equal to 0.5.

The coefficient of efficiency in Equations (8) and (9) is a function of the confinement geometry through the coefficient of horizontal efficiency, $k_{H}$, and the coefficient of vertical efficiency, $k_{V}$:

(10) $$k_{eff}=k_{H}\cdot k_{V};$$

(11) $$f_{1,eff}=k_{H}\cdot k_{V}\cdot f_{1}.$$

**Figure 30:**
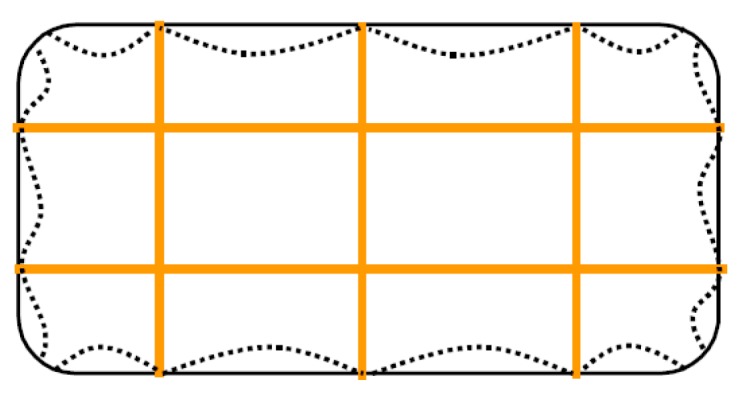
The cross-sectional area that is effectively confined in a column reinforced by both external wrapping and internal bars [66].

It is worth noting that equation 6 is the same expression used in Italian technical regulation [66] for the calculation of the design compressive strength in a masonry column confined with FRPs, in the case of combined use of discontinuous external wrapping and internal bars (Figure 30). The expressions used in [64] for $f_{1}$, $k_{H}$, and $k_{V}$, on the contrary, take into account the quincunx geometry of the CAM net.

Called $A_{m}$ the cross-sectional area of the confined masonry wall, the design vertical load assumed in [64] is equal to:

(12) $$N_{Rmc,d}=A_{m}\cdot f_{mcd}.$$

Therefore, contrarily to what prescribed in [66] for the FRP confinement, Marnetto and Vari do not apply any reduction factor to $N_{Rmc,d}$ when the confinement is provided by the CAM system. In other words, they neglect the difference between $A_{m}$ and the effectively confined cross-sectional area (Figure 30).

Moreover, in the absence of specific normative indications for masonry, Marnetto and Vari propose to calculate the ultimate strain of the confined masonry, $\epsilon_{mcu}$ (Figure 29), by amplifying the ultimate strain of unreinforced masonry, $\epsilon_{mu}$ (Figure 29), as for confined concrete [66]:

(13) $$\epsilon_{mcu}=0.0035+0.015\sqrt{\frac{f_{1,eff}}{f_{md}}}.$$

Lastly, the authors of [64] estimate the out-of-plane bending contribution of the vertical straps by using the formulas of the reinforced masonry, provided in [29].

**Figure 31:**
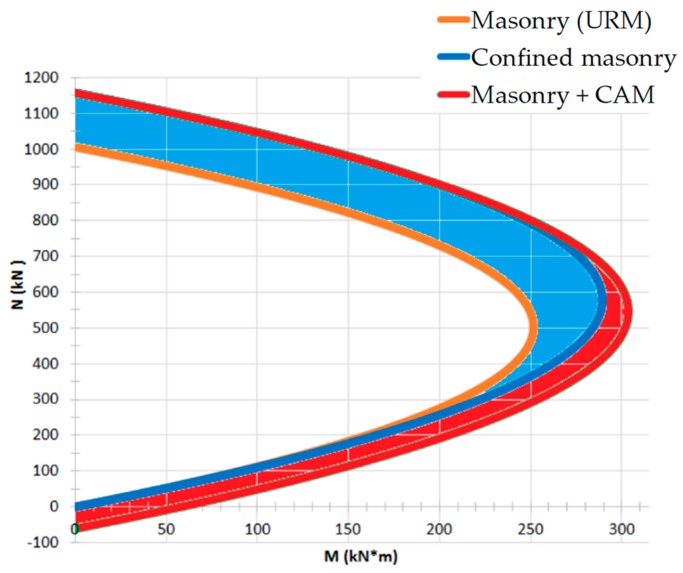
M-N interaction domain for a masonry wall reinforced with the CAM system [67].

In Figure 31, M and N are, respectively, the out-of-plane bending moment and the axial load resulting from Equations (6), (12), (13), and the formulas of the reinforced masonry, for masonry specimens 200 cm high and 40 cm wide ($f_{md}=1.48\;\mathrm{MPa}$) [67]. In particular:

- The orange plot is the limit domain for unreinforced masonry;
- The blue plot is the limit domain for confined masonry (only horizontal straps);
- The red plot is the limit domain for masonry reinforced by the CAM system (both horizontal and vertical straps).

From the comparison between the three limit domains in Figure 31, it seems—as claimed in [67] —that the CAM system significantly increases the resistant moments, in particular for high axial loads (blue area). In reality, the static analysis of Section 3.2 indicates that Figure 31 overestimates the effect of the horizontal straps. In fact, since the CAM system confines the masonry wall only in the transverse direction (Figure 24), $f_{mcd}$ increases due to the action of the transverse ribbons (through the Poisson effect), but not due to the action of the longitudinal ribbons (that is, the horizontal ribbons along the main dimension of the wall).

To be precise, the compressive stress in the longitudinal direction of the masonry wall does not increase due to the longitudinal ribbons, but increases, slightly, due to the impeded expansion in the longitudinal direction (Poisson effect) when the compressive stress increases in the transverse direction of the wall (due to the transverse ribbons). In other words, it is possible to evaluate the stress increase in the longitudinal direction (useful to calculate $f_{mcd}$) only by abandoning the simplified model with single masonry columns in Figure 28 and taking into account the mutual constraints between adjacent masonry units. In any case, the stress increase in the longitudinal direction due to the Poisson effect is lower than the stress provided by the longitudinal straps in the model with single masonry columns.

Therefore, it is reasonable to expect that equation 6 overestimates the value of $f_{mcd}$ supplied by the CAM system, thus leading to an overestimation of $N_{Rmc,d}$ in Equation (12). Moreover, the absence of any reduction factor in Equation (12)—not justified by the authors of [64]—may cause a further overestimation of $N_{Rmc,d}$.

In conclusion, the blue area in Figure 31 should be less wide. This ultimately means that the design criteria proposed in [64] underestimate the number of horizontal straps needed to increase the load-bearing capacity of a masonry wall.

## 5. Conclusions

This paper examines and discusses some of the latest active strengthening technique, useful for improving the seismic behavior of unreinforced masonry (URM) buildings. In particular, after having focused on the importance of providing the URM buildings with a box-type behavior, the authors analyzed the two main categories of active strengthening techniques that establish good connections between the structural elements of a building: the punctual and the continuous retrofitting techniques.

The comparison between the two main categories of active strengthening techniques showed that the continuous retrofitting techniques allow avoiding some typical problems of the punctual retrofitting techniques. In fact, unlike the punctual retrofitting techniques, the continuous retrofitting techniques do not involve excessive mass increases or stress concentrations at the anchorages. Furthermore, since the continuous retrofitting techniques use stainless steel ribbons, they also allow preventing problems of corrosion and chemical incompatibilities.

Lastly, after having exhausted their strengthening function (due to masonry failure), the continuous retrofitting techniques find a second use, since they begin to work as life-saving devices. In fact, the three-dimensional continuous nets of ribbons hold back the fragmented masonry, preventing any part of the structure from hitting people after failure. In short, structural failure does not mean structural collapse.

Therefore, the continuous retrofitting techniques seem to be very promising in the vast field of active strengthening techniques for URMs. However, since they are relatively new, their actual potential and even the strengthening mechanisms are, in part, still unknown.

With the aim of contributing to the knowledge of the actual strengthening mechanisms, the authors performed stress analyses in the Mohr/Coulomb plane for both the continuous retrofitting techniques known today: the CAM system and the Φ system. This static analysis represents the first attempt to explain how the CAM system and the Φ system modify the stress field in masonry walls for variable transverse stress, $\sigma_{T}$. In particular, the authors have shown that the actual strengthening mechanism of the CAM system is much more complex than the desired one, which should provide an additional hydrostatic state of stress to the masonry walls. In fact, the additional stress state given by the CAM system depends on the constraint conditions, that is, on the position in the wall of the retrofitted masonry unit. In any case, contrarily to what the researchers working on the CAM system believed up to now, it is neither a hydrostatic nor a tri-axial state of stress, except near the free ends and the openings of the masonry wall.

Moreover, from the comparison between the CAM system and the Φ system, the authors have found that:

- For masonry units of the lower stories, where the constraint degree is very high—at the limit, infinite—along the in-plane directions, the two continuous retrofitting systems perform almost the same way. In particular, both provide the maximum increase of the safety factor for low values of $\sigma_{T}$.
- For masonry units of the upper stories, where the constraint degree is low—but never equal to zero—along the in-plane directions, the effectiveness of the continuous systems depends on the additional transverse stress provided by retrofitting. In particular, for low values of $\sigma_{T}$ the Φ system is more effective than the CAM system in increasing the safety factor, for intermediate values of $\sigma_{T}$ the safety factor achieved after retrofitting depends on the single intervention and deserves further deepening and, lastly, for high values of $\sigma_{T}$ the maximum advantage in terms of safety factor is given by the CAM system.

For both systems of continuous retrofitting, it is not possible to increase $\sigma_{T}$ indefinitely: there is an upper limit value of $\sigma_{T}$ that cannot be exceeded, to avoid damage to the masonry. In the event of damage, however, a sliding plane originates that does not give rise to the collapse of the wall, as it is a vertical plane and the sliding takes place in the horizontal plane. The upper limit value of $\sigma_{T}$ depends on the lateral stress $\sigma_{L}$, that is, on the position in the wall of the retrofitted masonry unit. Therefore, in a multistoried building each story has its own upper limit value of $\sigma_{T}$.

One of the main consequences of the static analysis is that it is not possible to evaluate the stress field in a masonry wall retrofitted by the CAM system correctly, without taking into account the interactions between adjacent masonry units. In particular, the model with single confined masonry columns—used to date for the design of the CAM retrofitting system—leads to underestimate the number of horizontal straps needed to increase the load-bearing capacity of a masonry wall under static loads. Therefore, the model with single confined masonry columns is not a suitable sizing criterion for the CAM system.

This means that it is necessary to perform a more detailed stress analysis, in order to define new and more realistic design criteria for the improvement of the load-bearing capacity under static loads with the CAM system. Anyway, this does not affect the effectiveness as devices of safeguarding life, integrated into the structure, of the CAM interventions designed with the current criteria. Actually, the box-type behavior provided by the CAM system undoubtedly improves the seismic performance of masonry buildings, but the contribution of safeguarding life offered by the CAM system after a seismic event that caused serious damage to the masonry building is even more relevant.

## 6. Further Developments

A non-linear analysis could be very useful to integrate the static analysis performed in this paper. In particular, it could be useful to evaluate how the shape of the limit domains in the Mohr/Coulomb plane changes due to the strengthening effect of the steel ribbons on the masonry wall.

**Figure 32:**
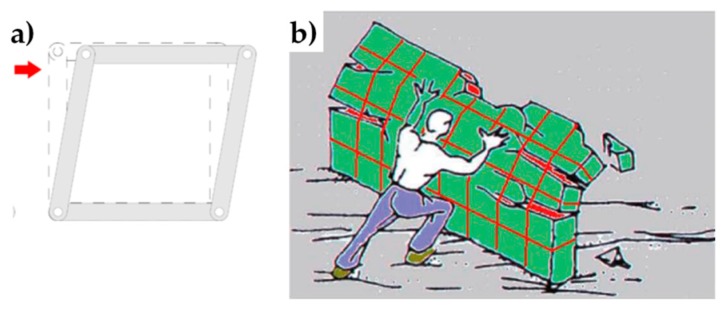
(**a**) Hinged mechanism between the ribbons of the rectangular arrangement of the CAM system; (**b**) Out-of-plane loading of a wall reinforced by the CAM system [52].

Furthermore, an extensive experimental program on the load-bearing capacity of masonry walls strengthened by steel straps at different pre-tension stresses could be useful to support the theoretical findings of the Mohr/Coulomb stress analysis. However, a careful analysis of the structural scheme made by the steel ribbons led the authors to consider another experimentation on the CAM system to be more urgent. In fact, as discussed in [49,69] for the CAM system, the structural scheme of the rectangular strap arrangement is labile both in the plane and the thickness of the wall, because the vertical loops form unbraced rectangular frame structures with hinged nodes (Figure 32a). While it is possible to find some solution to avoid the lability in the wall plane [49,69], the vertical rectangular loops in the wall thickness are not able to counteract the out-of-plane loads and sway laterally in any case (Figure 32b). Consequently, while both continuous retrofitting systems are effective in increasing the ultimate load of walls subjected to in-plane loading (for the CAM system, see for example [1,2]), they are almost at all ineffective in improving the out-of-plane strength of walls.

At the LiSG laboratory of the University of Bologna, the authors started an experimental program in order to investigate whether it is possible to modify or couple the basic scheme of the CAM system with other retrofitting systems, to increase also the out-of-plane ultimate load of the masonry walls. Experimental tests performed on a first set of three masonry specimens retrofitted by both the CAM system and CFRP (Carbon Fiber Reinforced Polymer) strips provided very promising results, which merit further investigation. See [68] for more details on the idea behind the experimental program and [69] for a summary of the early results. The results shown in [69] also provide evidence on the inability of the CAM system to improve the out-of-plane behavior, when applied alone.

## Figures and Tables

**Table 1:** Damage scale adopted in [23] to compute the typological seismic risk.

Label	Damage Level	Description	Masonry Buildings	Reinforced Concrete (RC) Buildings
DS0	No damage	—		
DS1	Negligible to slight damage	No structural damage, slight nonstructural damage	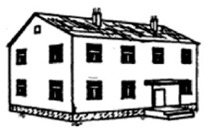	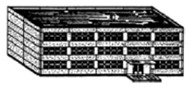
DS2	Moderate damage	Slight structural damage, moderate nonstructural damage	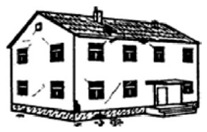	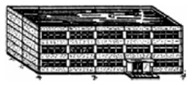
DS3	Substantial to heavy damage	Moderate structural damage, heavy nonstructural damage	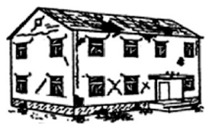	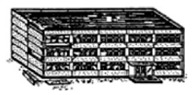
DS4	Very heavy damage	Heavy structural damage, very heavy nonstructural damage	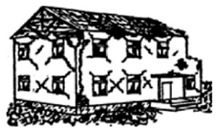	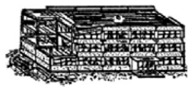
DS5	Destruction	Very heavy structural damage	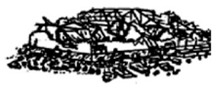	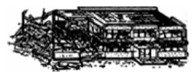

**Table 2:** Building typologies selected from those of [23].

Label	Building Class	No. of Stories
IMA1	Masonry—irregular layout—flexible floors—with tie rods and/or tie beams	1–2
IMA2	Masonry—irregular layout—flexible floors—without tie rods and tie beams	1–2
RMA2	Masonry—regular layout—flexible floors—without tie rods and tie beams	1–2
